# Perforated proximal jejunal gastrointestinal stromal tumor pT4N0M0 presenting with severe sepsis: A case report and literature review

**DOI:** 10.1016/j.amsu.2020.07.024

**Published:** 2020-07-20

**Authors:** Evander Meneses, Adel Elkbuli, Amanda Baroutjian, Mark McKenney, Dessy Boneva

**Affiliations:** a Department of Surgery, Kendall Regional Medical Center, USA; b Department of Surgery, University of South Florida, Tampa, FL, USA

**Keywords:** Jejunal GIST, Severe sepsis, Jejunojejunostomy, Perforated GIST

## Abstract

**Introduction:**

Gastrointestinal stromal tumors (GISTs) are mesenchymal tumors that occur along the alimentary tract, and are most commonly found in the stomach. Rarely, these tumors can occur in the small bowel, and when located in the duodenum or proximal jejunum, they may require challenging reconstruction of the alimentary tract. Patients with GISTs often present with non-specific abdominal pain or symptoms of obstruction, hemorrhage, and less commonly perforation.

**Presentation of case:**

A 46-year-old male presented to the hospital with a one-day history of left upper quadrant pain with fevers and chills. Physical examination was significant for signs of peritonitis, and laboratory results revealed leukocytosis and lactic acidosis. CT abdomen showed a large soft tissue mass in the proximal jejunum. In the operating theater he was found to have a perforated jejunal tumor. Pathology report revealed a 13cm GIST, pT4N0M0, Stage IIIa. He had an uneventful recovery and was discharged nine days after surgery.

**Discussion:**

Proximal jejunal GISTs are a rare entity and when present, perforation is unlikely. Pathological diagnosis of GISTs are relies on immunohistochemistry demonstrating c-KIT or CD34 positivity. The prognosis of GISTs are dependent on the size and the mitotic index. Definitive treatment of non-metastatic GISTs is R0 resection. When located in the duodenum or proximal jejunum, resection can be very challenging and may require clinical expertise in order to safely perform complex alimentary tract reconstruction.

**Conclusion:**

Further investigation is required in order to determine best practice management for patients who present with proximal GISTs.

## Highlights

• A rare presentation of proximal jejunal GIST as a necrotic perforated mass causing severe sepsis.
• Successful treatment of a pT4N0M0 (Stage IIIa) proximal jejunal GIST in the emergency setting with an uneventful hospital recovery.
• R0 resection of the proximal jejunal GIST with hand-sewn end-to-side anastomosis.

## 1 Introduction

Gastrointestinal stromal tumors (GISTs) are the most common mesenchymal tumors of the gastrointestinal tract [1,2]. These tumors originate from the interstitial cells of Cajal, which are located within the muscular layers of the alimentary tract [3]. GISTs have the potential to become malignant by an oncogenic mutation in the tyrosine kinase receptor (KIT) [4] and/or platelet-derived growth factor receptor-α (PDGFR-α) [5]. Size and mitotic index are the two most important prognostic features of GISTs [6].

Presentation of GISTs can vary from minor symptoms such as nausea and abdominal discomfort, to more severe manifestations such as acute abdomen. GISTs can also be found incidentally in some asymptomatic patients. They can occur anywhere along the GI tract, such as the stomach, small bowel, colon, omentum/mesentery, and esophagus [1]. While 50–70% of all GISTs occur in the stomach, proximal jejunal GISTs are a rare entity. The majority of literature available on proximal jejunal GISTS are case reports. Treatment of proximal jejunal GISTs are complex due to the anatomical position of the proximal jejunum and its close association with the ligament of Treitz. Treatment can vary from resection and primary anastomosis to resection and complex digestive tract reconstruction.

We present a case of an adult male who presented with severe sepsis and an acute abdomen secondary to a proximal jejunal mass rupture, which was successfully treated with a hand-sewn end-to-side jejunojejunostomy. We also reviewed current literature for this rare entity. This work has been reported in line with the SCARE criteria [7].

## 2 Presentation of Case

A 46-year-old male with hypertension and no prior surgical history presented to the emergency department (ED) with a one-day history of left upper quadrant abdominal pain. The patient reported having fevers and chills, and denied nausea, vomiting, hematochezia, weight loss, anorexia, and night sweats. He reported drinking alcohol socially and had no history of tobacco or recreational drug use.

Upon arrival, vital signs revealed a blood pressure within normal limits, he was febrile to 38.7 °C and tachycardia with a heart rate of 120 beats/minute. His physical examination was remarkable for left sided abdominal guarding and rebound tenderness. Laboratory analysis showed leukocytosis (20,000/L; normal 3600–11,000) and lactic acidosis (3.0 mmol/L; normal 0.4–2.0).

**Fig. 1:**
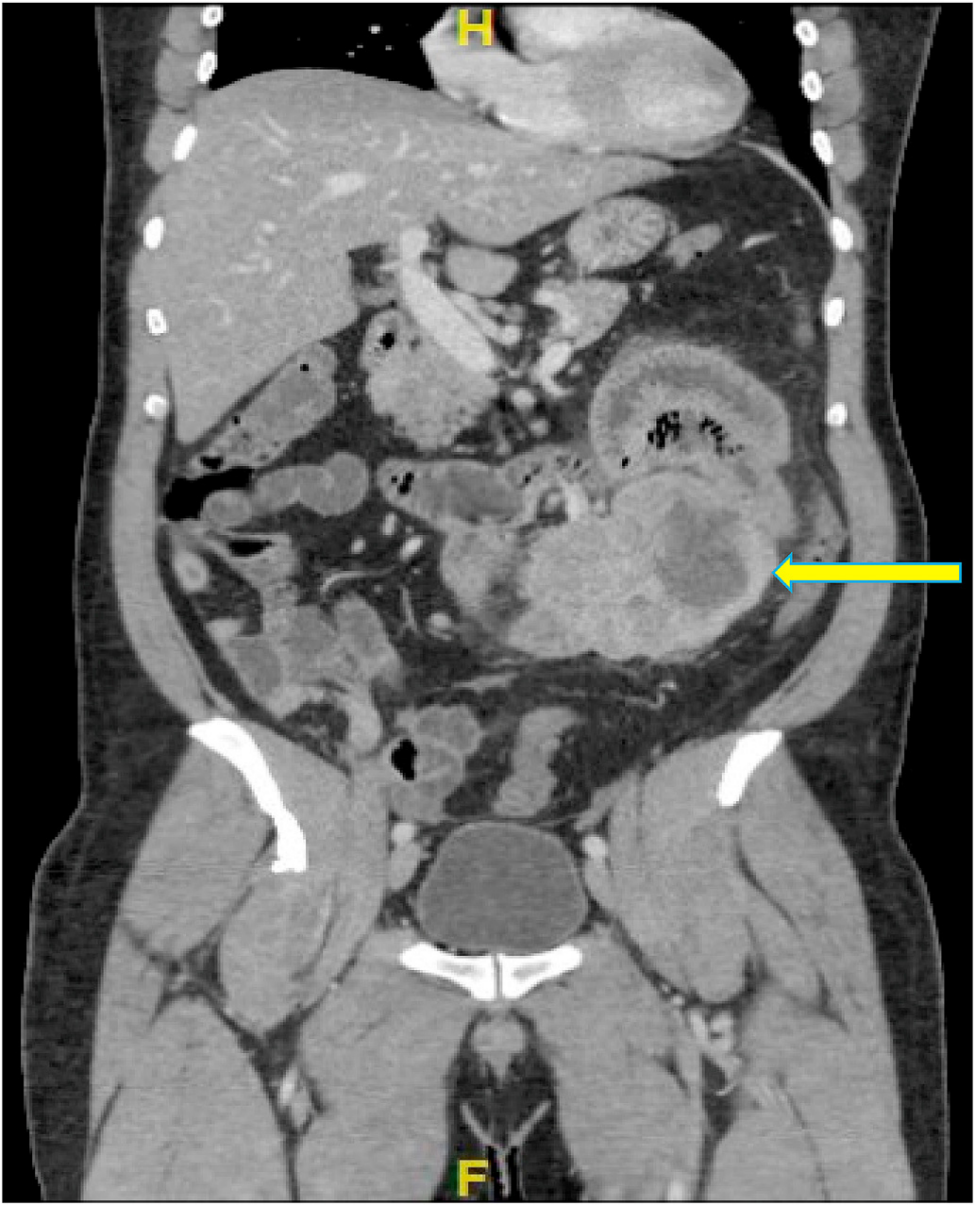
Coronal view of abdomen/pelvis CT scan showing the soft tissue mass, heterogeneous due to central necrosis. Proximal bowel dilation is seen due to the mass effect. The 3rd portion of the duodenum (horizontal portion) is seen dilated due to the mass. Compression of the adjacent bowel loops also is seen to cause a partial obstruction. Arrow pointing to mass.

**Fig. 2:**
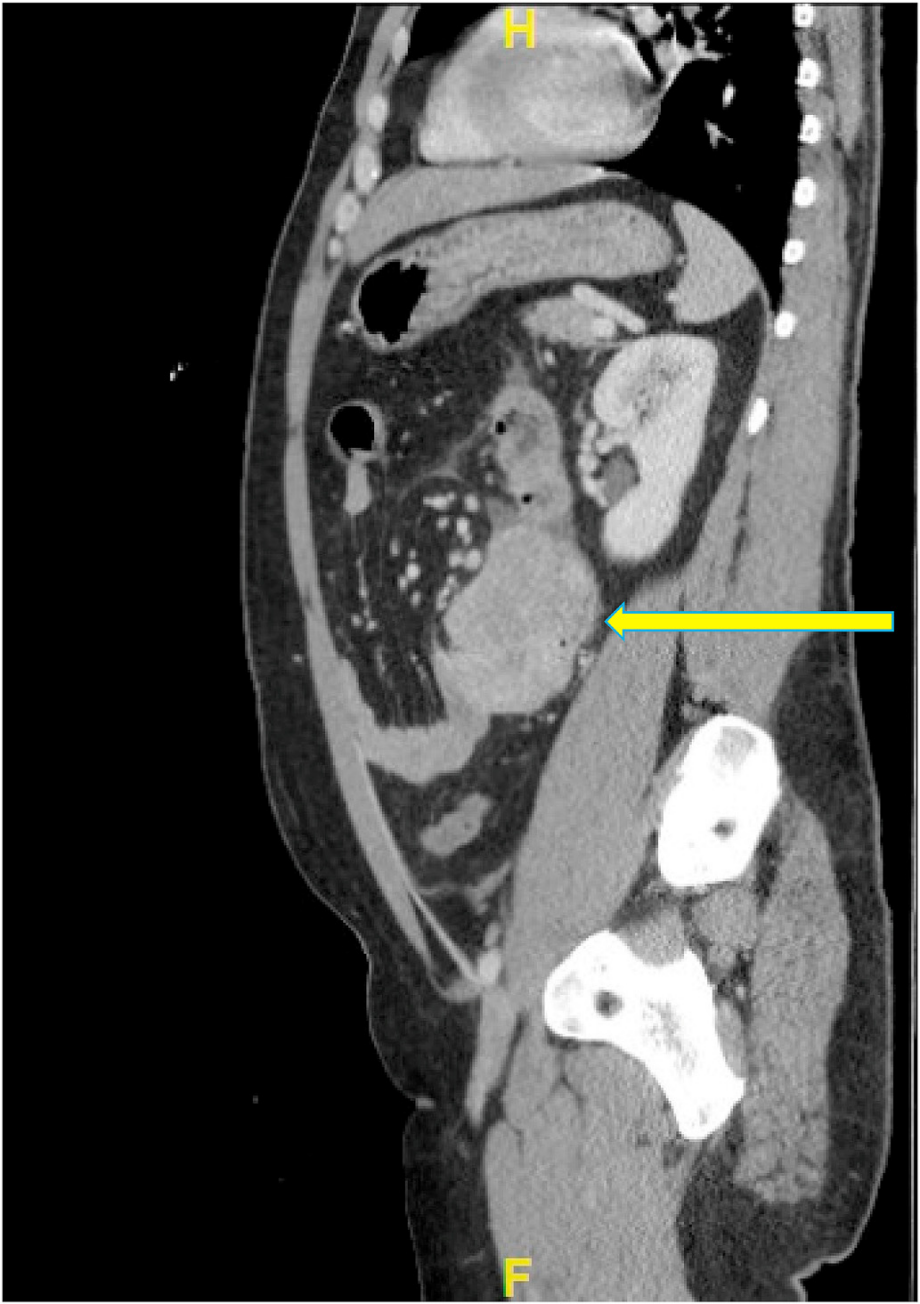
Sagittal view of abdomen/pelvis CT scan showing the GIST tumor – soft tissue mass seen in mid-abdomen. Arrow pointing to mass.

A computed tomography (CT) scan of the abdomen and pelvis with intravenous contrast revealed a soft tissue mass in the left upper abdomen measuring 13 × 6 × 7.5cm. The mass had central decreased density and necrosis, which appeared to result in compression of adjacent bowel loops and subsequent partial obstruction (Fig. 1, Fig. 2).

The patient was immediately started on broad-spectrum intravenous antibiotics in the ED. Due to his septic presentation of fever, tachycardia, focal peritonitis, leukocytosis, and lactic acidosis, the patient was taken to the operating room for an emergency exploratory laparotomy. Intraoperatively, a 13cm necrotic perforated mass was located 10cm from the ligament of Treitz. Significant contamination of the peritoneal cavity with purulent fluid was visualized. Following resection of the mass, the rest of the bowel and mesentery was thoroughly examined and did not reveal any additional nodules or lesions. A temporary vacuum wound dressing was placed due to patient's septic status. Subsequently the patient was transported to the intensive care unit (ICU) for adequate resuscitation and sepsis management with plans to return to the operating room for abdomen closure. Intravenous broad-spectrum antibiotics were continued post-operatively.

The preliminary pathology report revealed that the tumor was a low-grade GIST due to low mitotic rate. The final report showed that the tumor cells were positive for c-KIT and focally positive for CD34, while negative for pankeratin, desmin, S100 protein and smooth muscle actin (Fig. 5, Fig. 6, Fig. 7). The Ki-67 proliferation index was low at approximately 10%. Pathology also confirmed there was no lymphovascular invasion, no perineural invasion, absence of surgical margins involvement, a mitotic index of 2 per 50 HPF, sixteen lymph nodes negative for GIST, and no metastasis. This classified the tumor as pT4N0M0 per the American Joint Committee on Cancer's (AJCC) TNM classification [8]. The GIST was determined to be staged IIIa in accordance to the TNM classification and the low-grade mitotic rate (AJCC staging) [8].

On postoperative day one, the patient became afebrile and hemodynamically stable. His leukocytosis improved, and his lactic acidosis resolved after peaking at 9.9 mmol/L immediately after surgery. Following adequate fluid resuscitation and antibiotic treatment in the ICU, the next day the patient was taken to the operating room for an abdominal washout, establishment of continuity and closure of the abdomen. Due to the proximity of the proximal resected end of the jejunum to the ligament of Treitz by ~5cm, an end-to-side hand-sewn jejunojejunostomy anastomosis was performed and a feeding jejunostomy tube was placed 40cm from the jejunojejunal anastomosis. Postoperative imaging evaluation revealed patent surgical anastomosis.

**Fig. 3:**
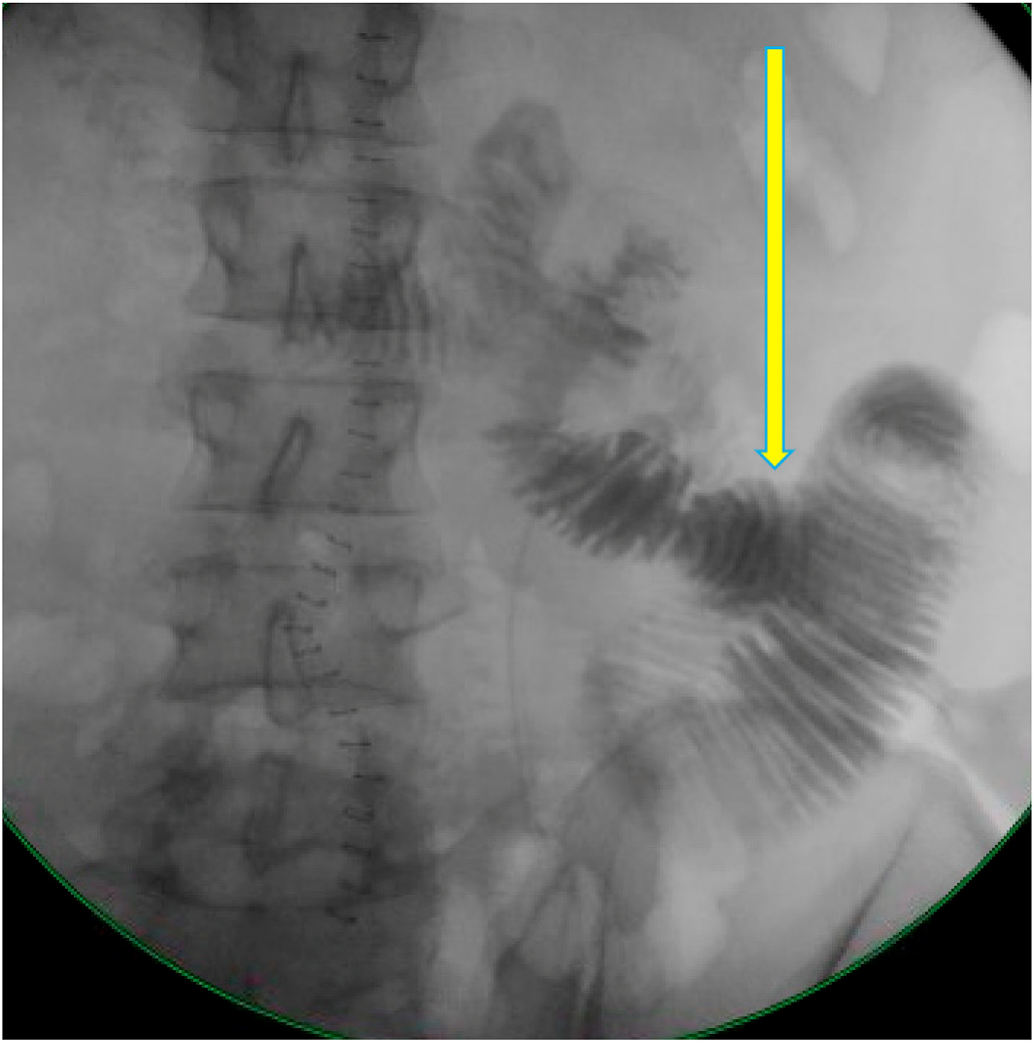
UGI SBFT – Upper GI and small bowel follow through done with oral contrast through the nasogastric tube showing contrast passing freely through the anastomosis without a leak. The side-to-side anastomosis is seen. The anastomosis is seen to be intact. Some jejunal dilatation is seen without SBO. Midline staples also observed. Arrow pointing to anastomosis.

**Fig. 4:**
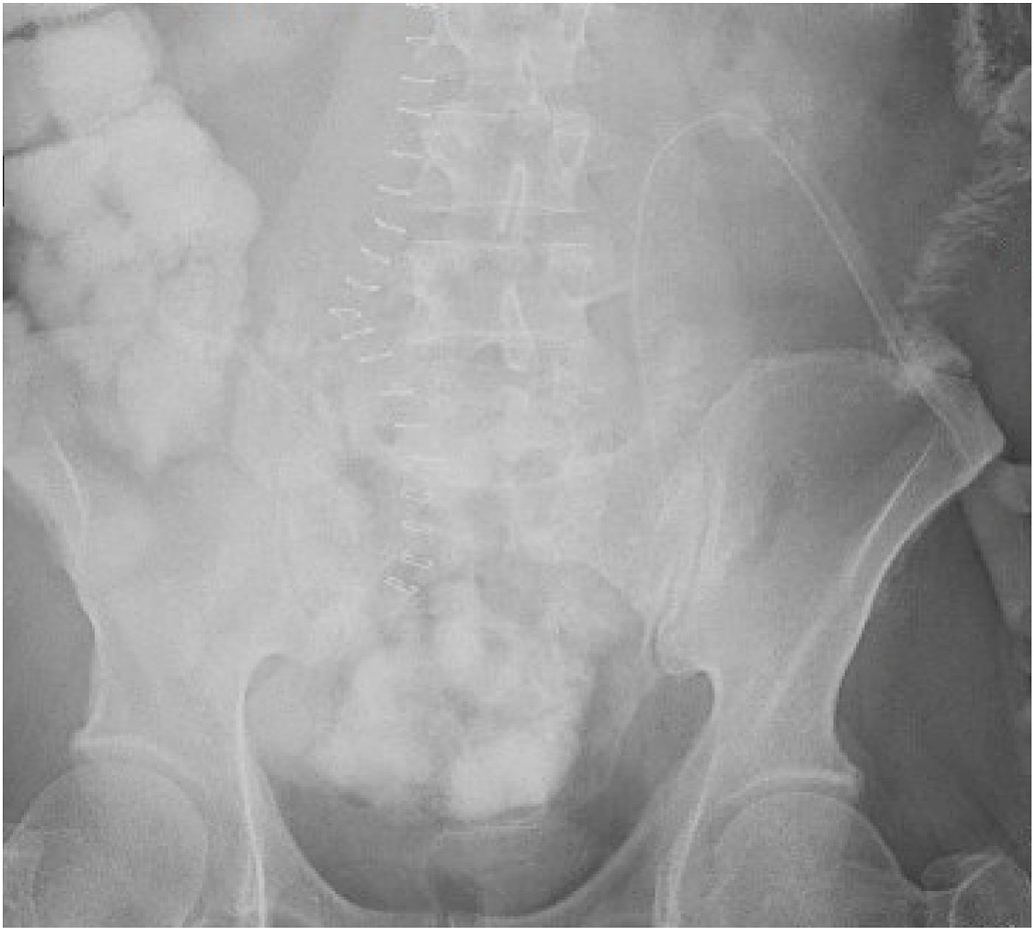
UGI SBFT – Upper GI and small bowel follow through series showing passing of PO contrast though the colon and into the rectum without obstruction. Jejunotomy feeding tube also seen and midline surgical staples are present.

On postoperative day two he was started on tube feeds through the jejunostomy tube and was tolerating jejunal tube feedings at his goal rate by day three. On day five, an upper GI small bowel follow through-study was performed, showing no anastomotic leak and an unremarkable stomach and duodenum with contrast reaching the colon at 3 h (Fig. 3, Fig. 4). On day six, he was started on a clear liquid diet which was advanced to a soft diet over the following 2 days.

**Fig. 5:**
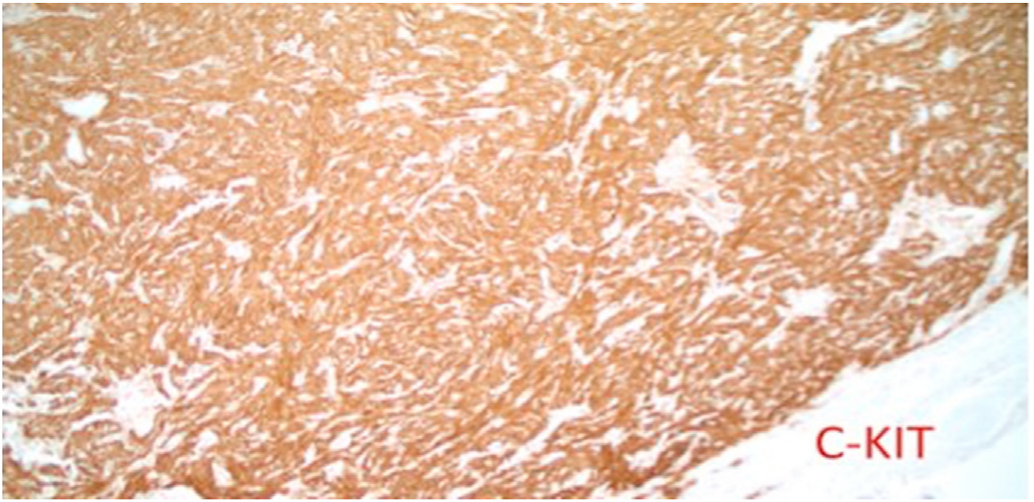
Immunohistochemical stain of proximal jejunal tumor showing positivity for c-Kit. This protein is a type of receptor tyrosine kinase, also called CD117.

**Fig. 6:**
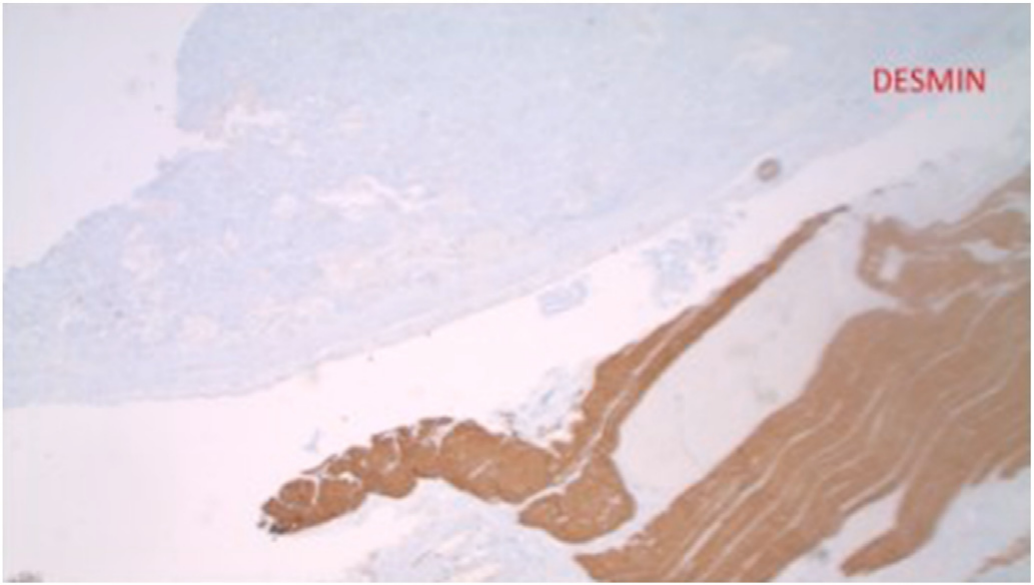
Immunohistochemical stain of proximal jejunum tumor negative for Desmin. This is a muscle-specific intermediate filament that can be seen positive in rhabdomyosarcomas.

**Fig. 7:**
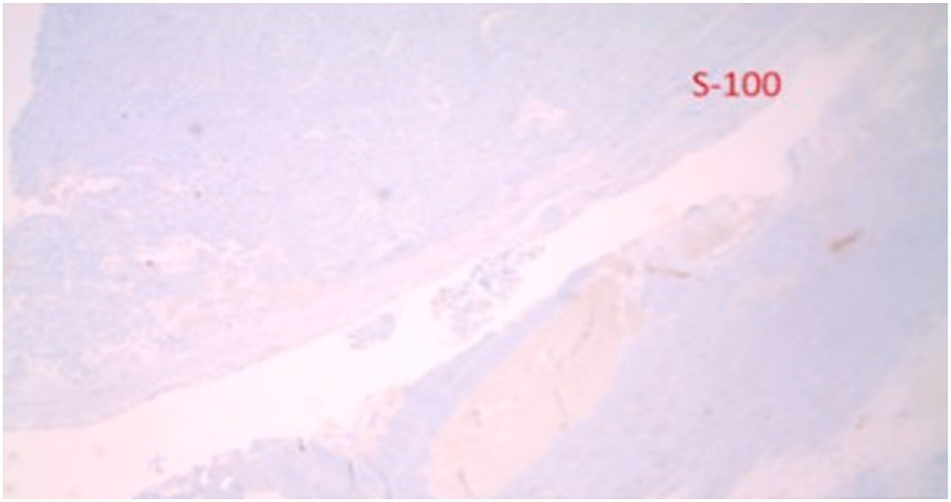
Immunohistochemical stain of proximal jejunum tumor negative for S100. This is a protein normally derived from neural crest cells and can be found in melanomas and schwannomas.

By day eight, his midline surgical incision was healing well with no signs of infection. He was tolerating PO diet, having bowel movements, afebrile and pain-free. On day nine, the patient was discharged in stable condition with PO antibiotics and further instructions to follow up with outpatient oncology. He was scheduled for an outpatient PET scan and a plan to start Imatinib therapy.

## 3 Discussion

Our patient presented with severe sepsis and was found to have a proximal jejunal necrotic and perforated mass that pathology later revealed to be a GIST. The patient required emergency surgery and due to instability was left open and in discontinuity on the index surgery. He was taken back to the operating room one day later after resuscitation for a hand sewn end-to-side jejunojejunostomy anastomosis and placement of a distal feeding jejunostomy tube.

The patient's presentation of a perforated GIST is rare. Current literature shows that jejunal GISTs usually present with non-specific abdominal pain or symptoms related to obstruction or hemorrhage. Seldom do GISTs in the jejunum cause perforation and peritonitis, and only a few cases have been reported in the literature [[9], [10], [11]].

**Fig. 8:**
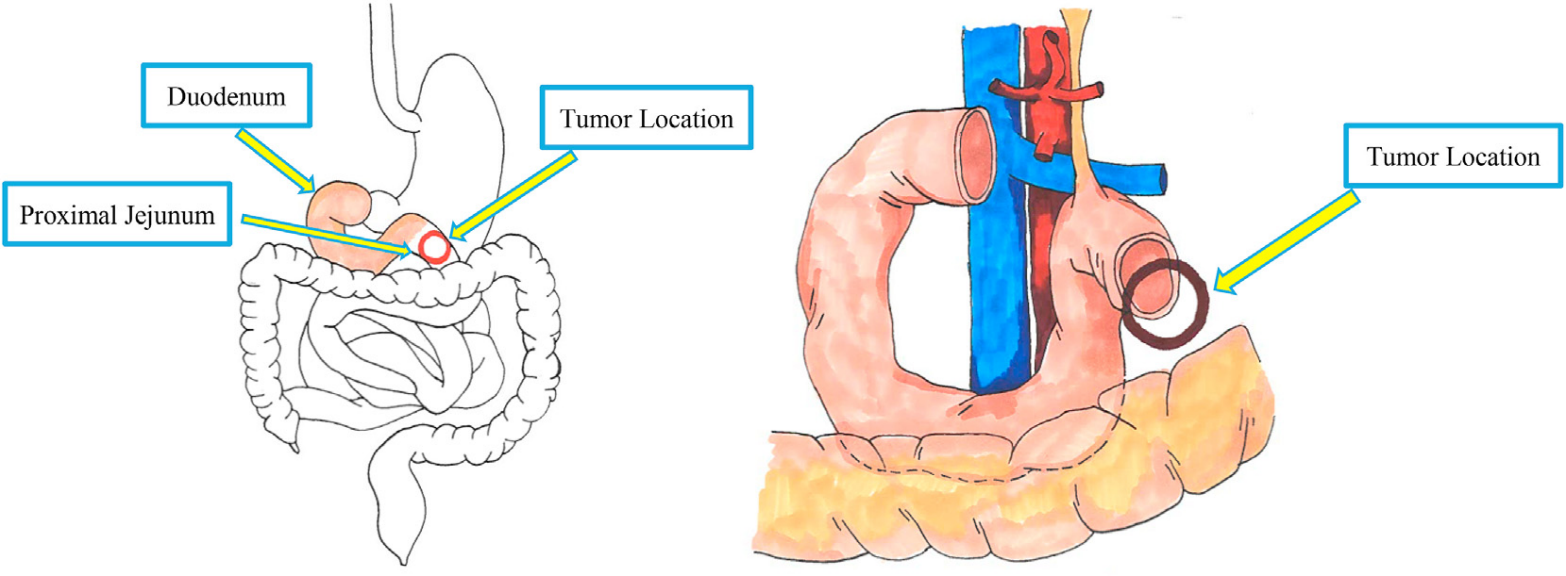
Duodenum seen – all 4 parts, the ligament of Treitz is observed. The soft tissue mass was located right after the ligament of Treitz making this tumor location anatomically more challenging/difficult location for resection and anastomosis. Area labeled jejunum is location of the tumor/soft tissue mass (5cm after the ligament of Treitz).

The location of the tumor attributed to the complexity of the case. While GISTs themselves are the most common mesenchymal tumor of the gastrointestinal tract, the majority are found within the stomach. The anatomical location of the tumor at the proximal jejunum made the creation of the jejunojejunostomy surgically challenging due to the proximity to the ligament of Treitz (Fig. 8). While a stapled side-to-side anastomosis would have been the ideal and preferred approach to create the anastomosis, it was not possible in this case due to the short length of the proximal jejunal limb, which prevented the use of a stapler to connect the ends of small bowel. In other circumstances where the jejunojejunostomy cannot be safely created due to the proximity to the ligament of Treitz, other surgical options such as a gastrojejunostomy, duodenojejunostomy or more complex bowel reconstruction must be considered, although they may result in higher rates of complications and worse outcomes [12].

Anastomotic healing was of utmost importance to our patient's recovery and favorable prognosis. An anastomotic leak or anastomotic failure would have been a devastating complication in the course of our patient's management. Our decision regarding the type of anastomosis to be created was based on the availability of only 5cm of proximal jejunum. Previous studies have shown no significant difference in anastomotic leak rates, morbidity or mortality between sutured versus stapled bowel anastomoses [[12], [13], [14]]. Today, while it is more common to perform a stapled anastomosis, the proximity of the proximal jejunal limb to the ligament of Treitz did not allow us to perform an adequately sized stapled side-to-side jejunojejunostomy. Instead, we performed a hand-sewn end-to-side jejunojejunostomy to avoid complications of anastomotic leak and stricture that have been observed in prior literature using an end-to-end anastomosis [15].

The decision to place a feeding jejunostomy tube 40cm distal to our anastomosis was due to the following reasons: to allow nasogastric decompression in the initial days following surgery, to prevent gastric secretions from placing undue stress on the new anastomosis, to circumvent gastric feedings from placing undue stress on the new anastomosis, and to allow early feeding to maintain a good nutritional status in order to promote anastomotic healing [[14], [17]]. Our reasons behind this decision were shown to be beneficial on day five when the upper GI series showed a patent anastomosis without a leak. In addition, the patient had a nasogastric tube, aiding in keeping the stomach decompressed from above. While a nasojejunal feeding tube could have been an alternative option to the open feeding jejunostomy tube, literature has shown a higher rate of dislodgment associated with nasojejunal feeding tubes compared to jejunostomy feeding tubes [16], and we wanted to avoid this risk in our patient.

The pathology upon immunohistochemical staining was classic for GISTs. GISTs are diagnosed when there is presence of KIT or CD34 positivity, although other stains such as DOG1 and smooth muscle actin (SMA) may also lead to the diagnosis of GIST(15). About 95% of GISTs are positive for KIT, 60–70% positive for CD34, 30–40% positive for SMA, 5% positive for S100 protein, 1–2% positive for desmin, and 1–2% positive for keratin(1). All of these markers were tested for in this case [1].

Pathology also revealed a 13cm GIST with R0 margins and mitotic index of 2 per 50 HPF. This is consistent with a pT4N0M0 GIST, or Stage IIIa GIST. According to AJCC, GIST staging is based on T (tumor size), N (lymph node invasion), M (metastasis) as well as mitosis (Table 1, Table 2) [16,18]. T4 indicates a tumor >10cm in greatest dimension; N0 indicates no regional lymph node metastasis; and M0 indicates no distant metastasis. Size and mitotic index are the most reliable prognostic factors of GISTs [6]. For GISTs ≤ 2cm with a mitotic index ≤5 per 50 HPF, the rate of recurrence is low [6,19]. Another risk of recurrence and progressive disease is the location of the GIST. For example, gastric GISTs >2cm and ≤ 5cm with a mitotic index ≤5 per 50 HPF have a 1.9% risk of progression after appropriate treatment, whereas a jejunal GIST with the same features have an 8.3% risk of progression [1]. To our knowledge, there is no mention in the literature of the outcomes of GISTs >10cm.

**Table 1:** TNM staging of GISTs.

T	N	M	Mitosis
T0 = no evidence of tumor	N0 = no regional lymph node metastasis or unknown	M0 = no distant metastasis	Low = ≤ 5/50 HPF
T1 = ≤2cm	N1 = regional lymph node metastasis	M1 = distant metastasis	High = >5/50 HPF
T2 = 2.1cm–5.0cm			
T3 = 5.1cm–10cm			
T4 = >10cm

**Table 2:** AJCC staging of jejunal GISTs.

AJCC Stage	TNM	Mitotic rate
I	T1 or T2	Low
	N0	
	M0	
II	T3	Low
	N0	
	M0	
IIIa	T1	High
	N0	
	M0	
	or	or
	T4	Low
	N0	
	M0	
IIIb	T2	High
	N0	
	M0	
	or	Or
	T3	High
	N0	
	M0	
	or	or
	T4	High
	N0	
	M0	
IV	Any T	Any rate
	N1	
	M0	
	or	or
	Any T	Any rate
	Any N	
	M1	

According to National Comprehensive Cancer guidelines, the gold standard treatment for GISTs without metastasis is surgical resection to achieve an R0 resection, which was achieved in this patient despite the complex location of the tumor. In some cases, neoadjuvant Imatinib therapy may be used post-surgery in those with high risk of recurrence [20]. As seen in a prior study, duodenal and proximal jejunal GISTs pose a complex problem due to anatomical complexity, however if an R0 resection is attainable then digestive reconstruction is unnecessary [21]. Had the tumor been unresectable, metastatic or recurrent, Imatinib would have been the primary treatment [14].

## 4 Conclusion

The presentation of a proximal jejunal perforated GIST causing severe sepsis and diffuse peritonitis is rare in medical literature and is associated with increased morbidity and mortality. Surgical intervention is required when diffuse peritonitis is present, however, in this case the complexity of tumor anatomic location precluded the usual management of small bowel resection with a stapled side-to-side anastomosis. Nevertheless, we were able to successfully perform a hand-sewn end-to-side anastomosis and safely resect the GIST without having to perform complex alimentary trach reconstruction, which resulted in an uncomplicated recovery. The patient was discharged home in stable condition with a plan to start his immune therapy and follow up with outpatient oncology. Future studies are needed in order to elucidate the safest and most appropriate management for septic patients who present with anatomically complex proximal jejunal GISTs.

## Ethical approval

This report was conducted in compliance with ethical standards. Informed written consent has been obtained and all identifying information is omitted.

## Funding

This research did not receive any specific grant from funding agencies in the public, commercial, or not-for-profit sectors.

## Author contributions

EM, AE, DB, MM– Conception of study, acquisition of data, analysis and interpretation of data. DB, MM – Management of case. EM, AE, AB, DB, MM –drafting of abstract, drafting of manuscript, critical revision of manuscript. AE, EM, DB, AB, MM – Approval of the final version for submission.

## Registration of research studies

This is a case report study.

## Guarantor

Dessy Boneva.

Mark McKenney.

## Informed consent

Informed written consent has been obtained and all identifying information is omitted.

## Provenance and peer review

Not commissioned, externally peer reviewed.

## Declaration of competing interest

No conflicts of interest.

## References

[1] NCCN task force report: management of patients with gastrointestinal stromal tumor (GIST)—Update of the NCCN clinical practice guidelines. https://www.nccn.org/JNCCN/PDF/GIST2007.pdf.

[2] Casali P.G., Abecassis N., Bauer S., Biagini R., Bielack S., Bonvalot S. (2018). Gastrointestinal stromal tumours: ESMO–EURACAN Clinical Practice Guidelines for diagnosis, treatment and follow-up. Ann. Oncol..

[3] Akahoshi K., Oya M., Koga T., Shiratsuchi Y. (2018). Current clinical management of gastrointestinal stromal tumor. World J. Gastroenterol..

[4] Hirota S., Isozaki K., Moriyama Y., Hashimoto K., Nishida T., Ishiguro S. (1998). Gain-of-function mutations of c-kit in human gastrointestinal stromal tumors. Science.

[5] Heinrich M.C., Corless C.L., Duensing A., McGreevey L., Chen C.J., Joseph N. (2003). PDGFRA activating mutations in gastrointestinal stromal tumors. Science.

[6] Fletcher C.D., Berman J.J., Corless C., Gorstein F., Lasota J., Longley B.J. (2002). Diagnosis of gastrointestinal stromal tumors: A consensus approach. Int. J. Surg. Pathol..

[7] Agha R.A., Borrelli M.R., Farwana R., Koshy K., Fowler A., Orgill D.P., For the SCARE Group (2018). The SCARE 2018 Statement: Updating Consensus Surgical CAse REport (SCARE) Guidelines. Int. J. Surg..

[8] (2018). Gastrointestinal Stromal Tumor Stages and Other Prognostic Factors.

[9] Memmi N., Cipe G., Bektasoglu H., Toydemir T., Kadioglu H., Bozkurt S. (2012). Perforated gastrointestinal stromal tumor in the jejunum: A rare cause of acute abdomen. Oncol. Lett..

[10] Feng F., Chen F., Chen Y., Liu J. (2011). A rare perforated gastrointestinal stromal tumor in the jejunum: A case report. Turk. J. Gastroenterol..

[11] Özben V., Çarkman S., Atasoy D., Doğusoy G., Eyüboğlu E. (2010). A case of gastrointestinal stromal tumor presenting with small bowel perforation and internal hernia. Turk. J. Gastroenterol. Off. J. Turk. Soc. Gastroenterol..

[12] Huang Y., Chen G., Lin L., Jin X., Kang M., Zhang Y. (2019). Resection of GIST in the duodenum and proximal jejunum: A retrospective analysis of outcomes. Eur. J. Surg. Oncol..

[13] Chassin J.L., Rifkind K.M., Sussman B., Kassel B., Fingaret A., Drager S., Chassin P.S. (1978). The stapled gastrointestinal tract anastomosis: Incidence of postoperative complications compared with the sutured anastomosis. Ann. Surg..

[14] Reiling R.B., Reiling W.A., Bernie W.A., Huffer A.B., Perkins N.C., Elliott D.W. (1980). Prospective controlled study of gastrointestinal stapled anastomoses. Am. J. Surg..

[15] Goulder F. (2012). Bowel anastomoses: The theory, the practice and the evidence base. World J. Gastrointest. Surg..

[16] Simillis C., Purkayastha S., Yamamoto T., Strong S.A., Darzi A.W., Tekkis P.P. (2007). A meta-analysis comparing conventional end-to-end anastomosis vs. other anastomotic configurations after resection in Crohn's disease. Dis. Colon Rectum.

[17] Han‐Geurts I.J.M., Hop W.C.J., Kok N.F.M., Lim A., Brouwer K.J., Jeekel J. (2007). Randomized clinical trial of the impact of early enteral feeding on postoperative ileus and recovery. Br. J. Surg. Incorp. Eur. J. Surg. Swiss Surg..

[18] Tosoni A., Nicolardi L., Brandes A.A. (2004). Current clinical management of gastrointestinal stromal tumors. Expet Rev. Anticancer Ther..

[19] (2018). Gastrointestinal Stromal Tumor Stages and Other Prognostic Factors.

[20] Huda T., Singh M.P. (2019). Gastrointestinal stromal tumors of small intestine. Surg. J..

[21] (2020). Soft Tissue Sarcoma.

